# Does prestige bias influence the recall and transmission of COVID-19-related information? Protocol registration for an experimental study conducted online

**DOI:** 10.1371/journal.pone.0281991

**Published:** 2023-02-23

**Authors:** Edwine Soares de Oliveira, André Luiz Borba do Nascimento, Washington Soares Ferreira Junior, Ulysses Paulino Albuquerque

**Affiliations:** 1 Programa de Pós-Graduação em Etnobiologia e Conservação da Natureza, Federal Rural de Pernambuco, Recife, Pernambuco, Brazil; 2 Laboratório de Ecologia e Evolução de Sistemas Socioecológicos, Departamento de Botânica, Universidade Federal de Pernambuco, Recife, Pernambuco, Brazil; 3 Universidade Federal do Maranhão, Bacabal, Maranhão, Brazil; 4 Universidade de Pernambuco, Petrolina, Pernambuco, Brazil; Max Planck Institute for the Science of Human History, Jena, Germany, HUNGARY

## Abstract

In epidemic and pandemic contexts, such as that of COVID-19, epidemiological changes are continuous, and many people do not have access to accurate, up-to-date information. In this context, social learning can be an advantageous survival strategy. We investigate whether people remember and communicate information attributed to someone prestigious more often than that attributed to family members, politicians, and people with experience in public health. The experimental phase will include a recall stage and an information transmission stage, which will be based on a fictitious text containing an opinion about a drug treatment for COVID-19. There will be four versions of the text, and each participant will be assigned one of these versions for the investigation. The participants will be instructed to read the fictional story and then complete a distraction exercise. Subsequently, a recall test will be performed, where they will be asked to recount the story as accurately as possible. The second stage of the experiment is aimed at testing the transmission of information where we will conduct a linear chain transmission experiment, where eight chains of four participants will be used for each story. They will be asked to write down their recollection of the material. This text will undergo spelling error correction and then be sent to the next participant in the chain through the platform. At the end of the experiment, there will be a self-reporting questionnaire for the participants; this allows for triangulation of the data.

## Introduction

In epidemic and pandemic contexts, such as that of COVID-19, there is a rapid rate of epidemiological change, and people do not always have access to accurate information [1]. In the early stages of such outbreaks, individuals need to make quick and important decisions on how to act amidst a large flow of incomplete and uncertain information [2]. Social learning can present itself as an advantageous survival strategy in such conditions [3].

The literature on cultural evolution has shown that, in the context of uncertainty, people need to select not only the information types (content) but also the information sources (models) [1–8]. Thus, in the context of a pandemic, learning from a model and attaining an above-average amount of information can be highly adaptive [3]. Recent evidence has shown that the source of information transmission in the pandemic context exerts considerable influence on people’s behavior [7, 9]. Therefore, different role models, such as family members [10], politicians [7], and the most prestigious individuals among the population [11], can influence how information is reproduced and transmitted. Of the existing set of role models that can influence information transmission, the prestigious type is very interesting. Henrich and Gil-White [12] argued that the evolution of prestige emerged through selective pressure to identify individuals within a group with “above-average” knowledge or skills, to learn from them. This would have led to information acquisition that increased aptitude. In other words, based on this theory, individuals who are more skilled within a certain domain knowledge tend to acquire prestige [12].

Individuals who enjoy prestige can be identified through factors such as personality, appearance, and material possessions. (first-order cues), as well as by the attention they receive from others (second-order cues), because prestigious individuals receive more attention within a population [12, 13]. According to the theory of the evolution of prestige, prestigious individuals can be influential even outside their domain of expertise [12].

In addition to influencing the transmission of information, the theory of evolution of prestige also implies that information from such individuals would be more memorable. The literature on adaptive memory has already provided evidence that certain pieces of information are remembered more than others [14–16]. In this sense, Henrich and Gil-White stated that, throughout the evolution of our psychology, a series of increasingly complex biases have shaped our minds [12]. However, the biases involved in the empirical studies on adaptive memory are mostly related to content or frequency of information [15, 17–19]; and to date, few investigations have been conducted within this theoretical background to determine the involvement of model biases. For instance, the study by Berl et al. [20], despite having been carried out with the aim of investigating the transmission of information, demonstrated that the prestige bias plays an important role in remembering. As predicted by Henrich and Gil-White [12] and with new evidence suggesting the role of prestige bias in human memory [20], it is possible that, overall, the prestige bias is active during both information transmission and recall in a context of uncertainty.

The COVID-19 scenario presents itself, then, as a large global experiment, with great potential for investigating how the prestigious role model may influence the recall and transmission of information and, consequently, influence human behavior. We intend to answer the following questions: [i] In a pandemic scenario, does a prestigious role model exert greater influence on information transmission than political and family role models and role models with expertise? [ii] In a pandemic scenario, does a prestigious role model elicit more information recall than political and family role models and role models with expertise?

## Hypotheses

**Hypothesis 1**: The transmission of information related to COVID-19 is strongly influenced by a source who possesses prestige.

*Prediction*: Information related to COVID-19 that is attributed to someone with prestige (digital influencers) is transmitted more often than the same information attributed to political role models, role models with public health expertise, and family members.

According to Henrich and Gil-White’s [12] theory, prestige is based on the merit that other people confer on certain individuals, which promotes greater admiration from others and a desire to be close. The "status" involved in this is proportional to the amount of attention received. Thus, as an individual receives more attention, their status will be higher [10]. The assumptions of the theory are as follows: [i] qualified individuals have a higher status; [ii] older individuals tend to obtain more prestige than younger individuals; [iii] individuals with perceived skill/knowledge receive privileges and are exempted from certain social obligations; [iv] people preferentially mirror qualified/successful individuals; [v] prestigious individuals are influential even beyond their domain of expertise; and [vi] prestigious individuals are more memorable.

Jimenez and Mesoudi [13] gathered existing evidence in the literature on prestige bias and added to their initial predictions, including the following: [i] prestigious individuals tend to be successful in domains that are currently important to a social group or that were valued in the recent past; [ii] prestigious individuals only achieve social influence when their domain of prestige is currently valued by a social group; [iii] the positive association between perceived success and prestige will be greater than the positive association between actual success and prestige; [iv] a more positive correlation between prestige and success indicates that more people will employ social learning with prestige bias; [v] prestigious individuals will be mirrored more often when the variation in knowledge/skill within a group is larger; [vi] prestige bias across domains occur more often when cues are not as evident within the domain tested than when those cues are clear; [vii] prestige bias across domains occur when there are general traits, which make people successful across domains [13].

To identify individuals who hold prestige and thus select the information to be reproduced, learners can evaluate different individuals’ competence within a given domain and then choose the best individual to follow. However, this can be very time-consuming and costly for survival. For this reason, learners use “shortcuts” called prestige cues to make inferences about the individuals’ characteristics such as appearance, personality, generosity, material possessions, (first-order cues), or they rely on the behavior of other social learners (second-order cues). Both can be useful for acquiring potentially adaptive information; conversely, they can also lead to unhelpful or maladaptive behavior [13].

First-order cues are based on the fact that these shortcuts generally have a positive association with competence in important domains. Nevertheless, this identification is subjective, as it depends on the social learners’ values as well as their social group. They are less cognitively demanding and, therefore, quite advantageous, but they may not be fully reliable. In contrast, second-order cues, are related to the fact that individuals pay more attention to, want to be near, and mirror individuals who are competent. Consequently, social learners can use other individuals’ behavior to select which role models to learn from. The advantage of these cues is that they are more difficult to falsify (unlike first-order cues) and can be updated regularly [13].

Recently, Berl et al. [21] developed a valid, reliable scale to assess individual prestige. The scale is based on three central components: position, reputation, and information. Position concern a given individual’s place in the social hierarchy, and it is based on three dimensions: wealth, high social status, and power (or influence). Reputation involves the individual’s social opinion and esteem; the latter represents the overall social evaluation of an individual in a particular position or function, which usually involves social and cultural values. Finally, information is composed of items relative to education and intelligence, or the value that society gives to those who demonstrate more knowledge about a certain subject [21]. This last component is also based on Henrich and Gil-White’s [12] theory that individuals can gain prestige by presenting desirable skills and knowledge within a given social group.

Prestige bias allows individuals to acquire information quickly and is less costly in a new environment, thus increasing the chances of acquiring potentially more adaptive information [12]. Atkisson et al. [22] investigated this through an experiment in which participants designed arrowheads and tried to maximize hunting success; this was measured in caloric return. Their main findings revealed that participants learned preferentially from prestige models, defined as models that others spent more time looking at, and that prestige information and success-related information were used to the same degree. Thus, their results support the important role that prestige bias plays in social learning [22].

Moya et al. [1] noted that in a context such as the COVID-19 pandemic, in which epidemiological and social changes were rapid and abrupt, the source transmitting information can exert great influence on the information that people will reproduce. Recent evidence shows us that the existence of social bubbles on social media platforms such as Twitter allows for false and true information about COVID-19 to be shared in the same proportion; additionally, this conformity may be associated with ideological issues and the person transmitting the information [9]. In light of this information and the theory of the evolution of prestige, we believe that people will select sources with social prestige to reproduce and transmit information about COVID-19.

**Hypothesis 2**: Recall of information related to COVID-19 is strongly influenced by a source that possesses social prestige.

*Prediction*: Information related to COVID-19 attributed to someone of prestige (digital influencers) is transmitted more often than the same information attributed to political role models, role models with public health expertise, and family members.

The way our memory works demonstrates how our ancestral psychology has evolved [23]. In the theory of prestige evolution proposed by Henrich and Gil-White [12], human memory has evolved into an increasingly well-organized and specialized set of biases. Prestige biases emerge from this evolved social learning psychology and may be influential, along with the mode of transmission, in information memorization [12]. For example, the empirical study of cultural transmission by Berl et al. [20] found evidence that prestige bias has significant effects on the information that people can remember. However, it did not examine whether this is also the case in unstable settings [such as the COVID-19 setting]. Jimenez and Mesoudi [24] also tested whether information provided by prestige models is better remembered over generations than information provided by low-prestige sources. The authors did not find sufficient evidence to support this hypothesis. Based on these results, and unlike the theory of prestige evolution, they assumed that it would not affect the recall of information along the transmission chains [24]. However, besides not being a specific memory experiment, the authors addressed something stable in daily life (the use of the tablet in the school environment). Furthermore, they also acknowledged the possibility that their manipulation of prestige did not adequately cover the way prestige works in everyday life. Therefore, it is unclear whether the result derived is due to the domain studied or due to methodological limitations, or whether prestige actually has no effect on recall.

We intend to properly control our experiment by clearly explaining to the participant the characteristics that confer prestige on the source of information. These characteristics are based on the scale and prestige cues developed by Berl et al. [21] to measure individual prestige. We will also use political, knowledgeable, and familiar sources who do not have prestige but are role models who exist in the real world. This will allow us to investigate whether prestige can, in fact, influence the flow of information within a pandemic context.

## Experimental design

### Ethical aspects

This project was approved (number 5.329.534, in 04/04/2022) by the Ethics Committee for Research with Human Beings at the Federal University of Pernambuco, as required by the current legislation (Resolution No. 466 of December 12, 2012). All volunteers, over eighteen years of age, in the study will be asked to write out an online authorization regarding the Free and Informed Consent Term, as instructed in resolution No. 466/12 of the National Health Council. There will be no payment for the participation of each volunteer; however, at the end of the research, a reward will be available for the full completion of the experiments.

### Project

We intend to investigate whether people remember and transmit information more often when attributed to a prestigious source, in a pandemic scenario. We will use an online platform to carry out an experimental study, without the need for follow-up. The experiment will be conducted in two distinct stages, a recall stage as well as a stage for transmitting the information, both of which will be based on a fictitious text offered to the participants. This text will contain information about the use of a drug for COVID-19. There will be four versions of the text, one for each role model to be tested. Each version will consist of the same information, but only the role model attributed to the information being transmitted will change.

Since the experiment will be carried out online, there will be no need for blinding, on the part of the researchers, in carrying out the experiment and analyzing the results. More information is provided below about the selection of information along with a detailed procedure of the experiments. All data from the survey will included as supplementary material in the final articles.

### Sample size

**Fig 1:**
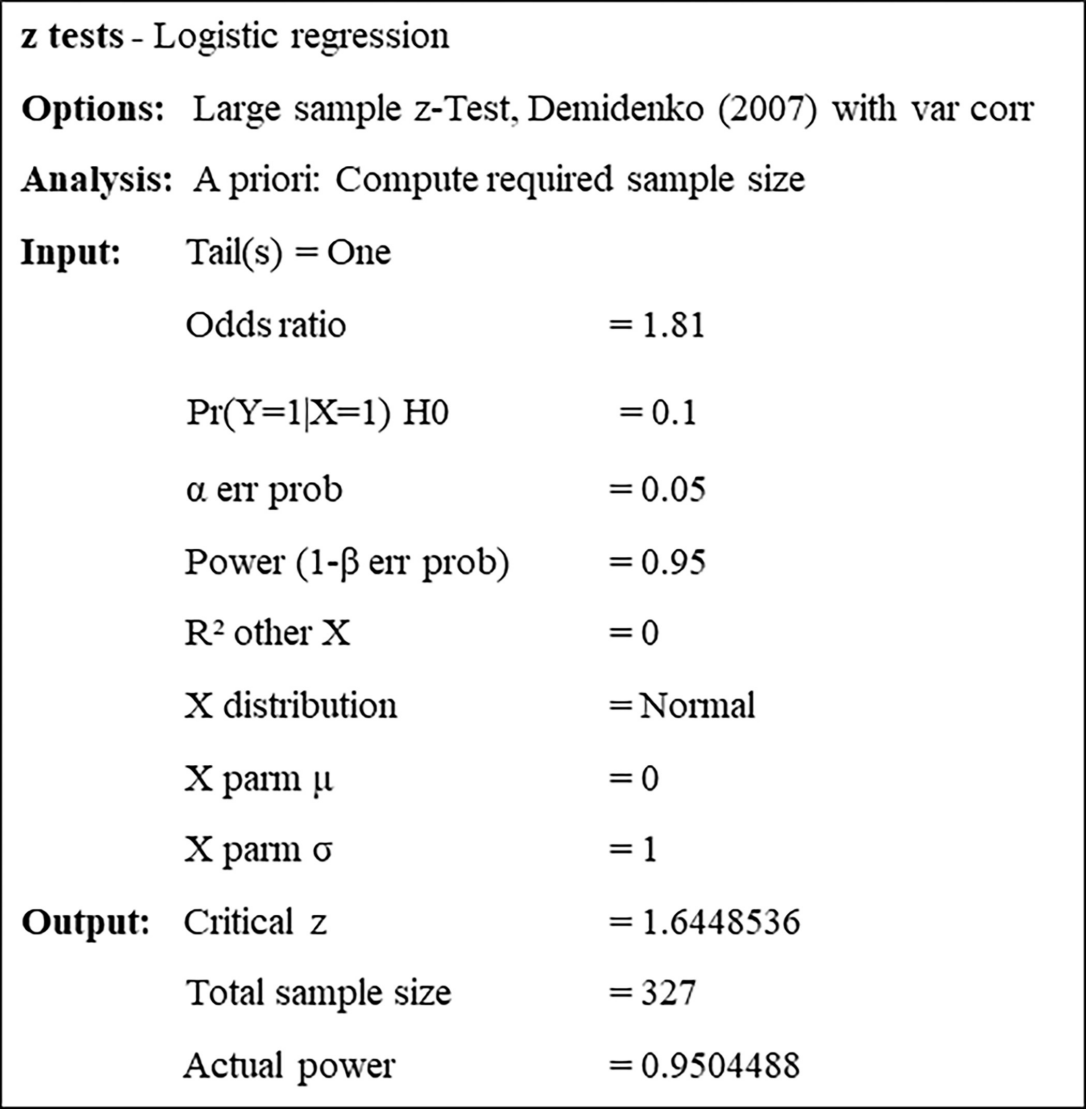
Parameters used to calculate the sample size based on a power analysis.

Individuals aged 18 years and older with access to the Internet will be selected to participate in the research. The recruitment of volunteers will be carried out through the dissemination of the survey on social networks such as Instagram, Twitter, Facebook, WhatsApp, and E-mail. To determine the sample size for the recall stage, we used the G*Power 3.1.9.7 software [25]. We conducted an *a priori* analysis to determine both the sample size and its power. As a basis for the study, we took the effect size (Odds ratio = 1.81) in a study by Bonin et al. [26] as it is the most recent study that brings memory-related variables and employs the same statistical test that we propose to use. We calculated a sample size of 327 people with an explanatory power of 95% and z = 1.64. The default for Pr(y = 1|x = 1)H0 in the G*Power software is 0.2 (i.e., a 20% probability of the event occurring if the null hypothesis is true). However, we use 0.1 to obtain an even more reliable sample size for the phenomenon we are investigating. We consider an α of 0.05, a Power (1-β err prob) of 0.95, R^2^; = 0, and normal distribution. Fig 1 presents the parameters used for this calculation.

The calculation for the transmission stage was based on the same parameters. However, as this stage will be conducted in transmission chains of four people each, we divided the total number of 327 people by 4 and rounded the result to the nearest whole number, arriving at a total of 21 chains per text used and 336 participants (an increase of 9 people from the calculated sample).

#### Selection of the information used

Since information about COVID-19 has been in wide circulation throughout the population for almost two years, we will not use data pertaining symptoms and actual forms of prevention, as there is a risk of bias. Therefore, we will create fictitious information appropriate to the context. To select our prestigious role model, we considered the characteristics described in the scale developed by Berl et al. [21] to assess how people attribute prestige. These characteristics are based on social status and esteem, influence, and knowledge of a certain subject. As social media played an essential role in the pandemic scenario, mainly due to isolation measures [27], and personalities such as digital influencers gained a lot of notoriety, they serve as ideal examples for the prestigious role model. In this case, we consider digital influencers to be people who receive a significant amount of attention (the more followers they have, the greater their engagement and popularity) and have a prominent social status, expertise within their niche, and influence beyond that niche.

For the decision of the other models to be used (to be compared with the prestigious role model), we selected a role model who had knowledge on the subject of public health, but no prestige, to assess whether prestige is in fact more influential than expertise, in this scenario. For the other role models, we used a study carried out by Hornsey et al. [28], where it was stated that there are psychological roots behind our attitudes of accepting or rejecting scientific information. Six roots are then described: ideologies, vested interests, conspiracy worldviews, fears and phobias, expression of personal identity, and social identity needs. Conceptually, these roots are distinct; in practice, however, they can overlap [28]. We selected models that may have behaviors and attitudes that reflect the overlapping of these roots, such as politicians, who share the same ideologies and interests; family members, who, along with previous roots, also have the same worldviews, while also possibly validating opinions related to personal fears and phobias.

Furthermore, empirical studies have shown how politicians’ opinions are taken into account in decision-making during a pandemic [7]. Recent studies also reveal that family care is a human priority [10], so it is quite plausible for people within the family to act as role models.

We will ensure that the selected models do not contrast with the ideologies of each participant, they are unnamed, and they are in line with the political positions and personal tastes of each reader, which is clear in the texts (Table 1).

**Table 1:** Models used in the elaboration of the text to carry out the recall experiments and transmission of information about COVID-19.

Role Models	
Prestigious	Digital influencer with many followers and considerable influence that the participant follows and admires
Political	Politician
Experience/Knowledge	Physician
Parental	Family member (one who is closest to the participant)

### Elaboration of the texts

The texts will contain a brief contextualization of the pandemic scenario; then, a particular opinion about the use of the drug Postex in the treatment of COVID-19 will be reported and attributed to one of the role models. By attributing the opinion to the role models, we will describe the characteristics that may confer prestige onto them. In this way, we will be able to determine whether the prestigious role model influences the retention and transmission of information. The stories have approximately the same number of words, ranging from 123 to 129 (S1 File).

All texts have the same structure; first, the pandemic context; next, a model with an opinion on the use of the medication. The number of central propositions referring to each of the models is also equal (Table 2).

**Table 2:** Central propositions used in the story texts for the experiments on recall and transmission of information about COVID-19.

Central Propositions	
Contextualization	Pandemic context
Proposition 1	Characterization of the role model
Proposition 2	Support for use of the drug
Proposition 3	Decrease in the number of deaths
Proposition 4	Decrease in the number of hospitalizations
Proposition 5	Assertion that use is safe
Proposition 6	Success rates exceed failure rates
Proposition 7	Postex should be used

All texts will be randomized so that they have the same chance of being assigned to participants.

## Data collection procedure

### Recall stage

The first experiment aims to test the hypothesis on the recall of information related to COVID-19 when it is presented by a prestigious role model. The entire experiment will be carried out online, through a platform specifically developed for research of this nature, by the Laboratory of Ecology and Evolution of Social-ecological Systems at the Federal University of Pernambuco.

**Fig 2:**
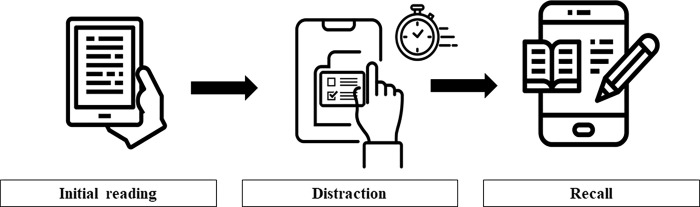
Recall experiment to be performed in Experiment I. Figure based on the protocol adapted from Silva et al. [18].

The protocol, adapted from a study by Silva et al. [19], involves offering reading material to a participant, immediately followed by a distraction activity, and then conducting a surprise recall test of the information that was read (Fig 2).

Upon clicking the research link, the participant will see a brief presentation of the research, followed by the consent and authorization forms to publish the data (referring to the TCLE). After accepting to participate, the volunteer will go to the next page, which will contain a form (S2 File) to fill out their data (gender, age, education, occupation, state where they live), region (urban or rural), education level, income, political inclination, and religion. The participants will then be directed to the next page, where there will be an alert, “*Now you will be asked to read a short text*. *Please read carefully as some questions will be asked later*.” after which the participant will be directed to one of the texts previously described. The texts will be randomized by the platform’s algorithm before being made available to each participant. After reading the text, they will be directed to a short attention test, which will verify whether the participant is paying attention to the experiment and also serve as a distraction activity. The test will consist of identifying certain figures (S3 File). Each page will have a two-minute time limit, which is an important step, as it ensures a standardized time for all the participants.

Finally, the volunteer will undergo a recall test, where they will be instructed to recount their respective reading text as accurately as possible. The instructions are as follows: “*Write down what you can remember about the text you read previously*. *Be as accurate as possible and don’t worry if you cannot remember all of the information*. *You have 10 minutes to complete this activity*.” At the end of the activity, a self-reporting stage of multiple-choice questions (S4 File) will follow with: [i] Who do you trust to seek information about COVID-19? (Participants can choose more than one option, and the alternatives will be health professionals, family members, friends, politicians, among other day-to-day personalities) [ii] What is your biggest source of information about COVID-19? (They can choose more than one option, and the alternatives range from online to physical media, such as newspapers and magazines) [iii] Have you been affected by COVID-19? (The options here will be yes or no, if yes, they will be asked to what extent they were affected) [iv] Has any member of your family been affected by COVID-19? (The options are the same as for the previous question) [v] Have you been vaccinated against COVID-19? The objective of this step is to perform data triangulation between the model that the person claims to follow and the factor that influenced the recall. Upon completion, the participant will see a message of acknowledgment and appreciation and will then be removed from the platform.

### Transmission stage

The second experiment to be carried out aims to test the hypothesis referring to the transmission of information related to COVID-19: specifically, on whether the presence of a prestigious role model favors the transmission of information over successive generations. Similar to the first experiment, this one will be carried out online, through a platform specifically developed for research of this nature, by the Laboratory of Ecology and Evolution of Socioecological Systems at the Federal University of Pernambuco.

**Fig 3:**
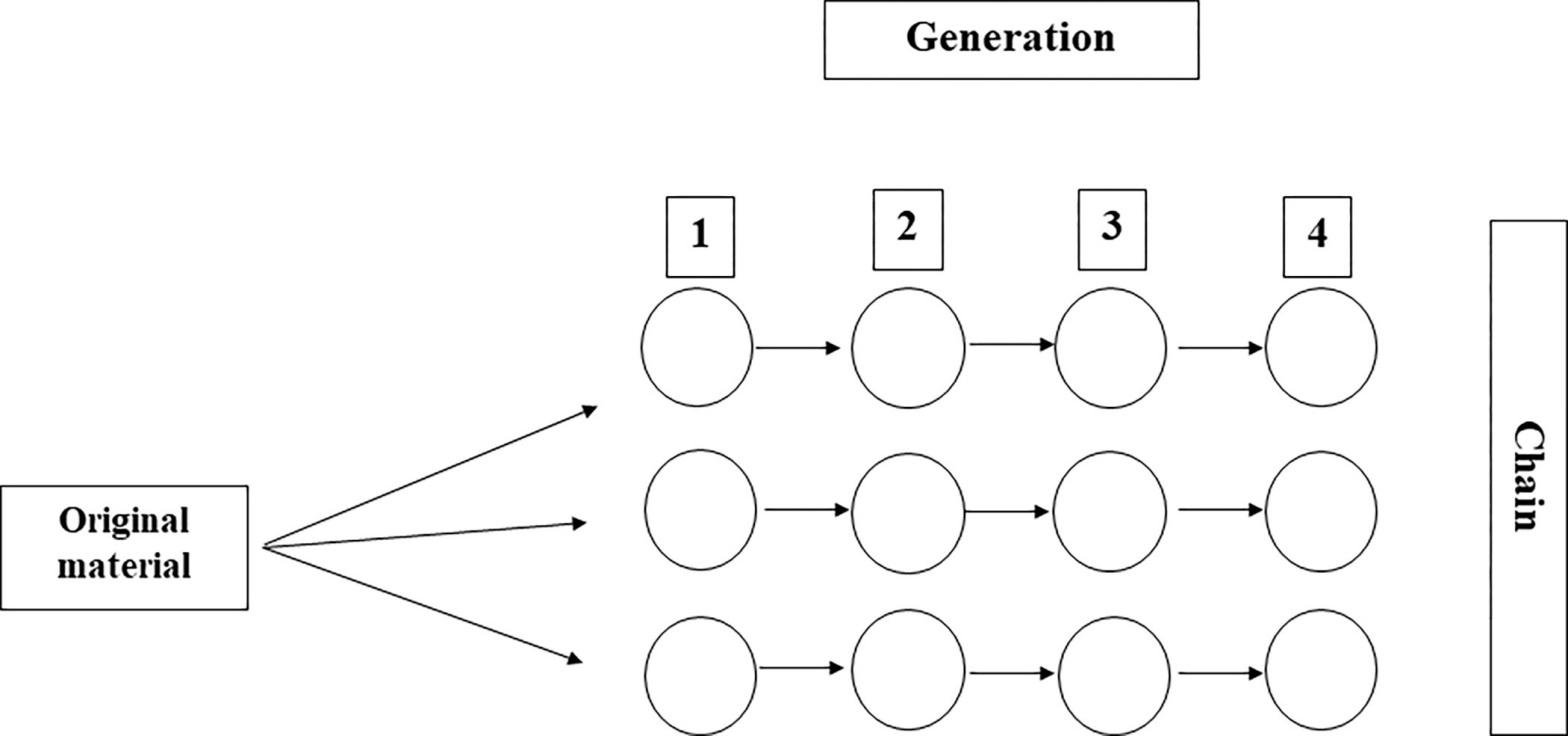
Representation of a linear chain transmission study [30].

A linear transmission chain experiment will be carried out, a method originally developed by Bartlett [29], which has been used in studies of cultural evolution over the years. It allows for the simulation of information transmission along a chain of individuals and occurs as follows: the first participant in the chain reads the material and, after a distraction task, is asked to recall the material read. In the original method, the resulting recall is then passed to the second participant, who does the same, and this continues along the chain (Fig 3). We will test 16 chains of four participants (representing four generations) for each proposed condition, totaling 64 chains.

The initial phase of this experiment will be similar to the first experiment, from clicking on the research link right up until the surprise recall test.

**Fig 4:**
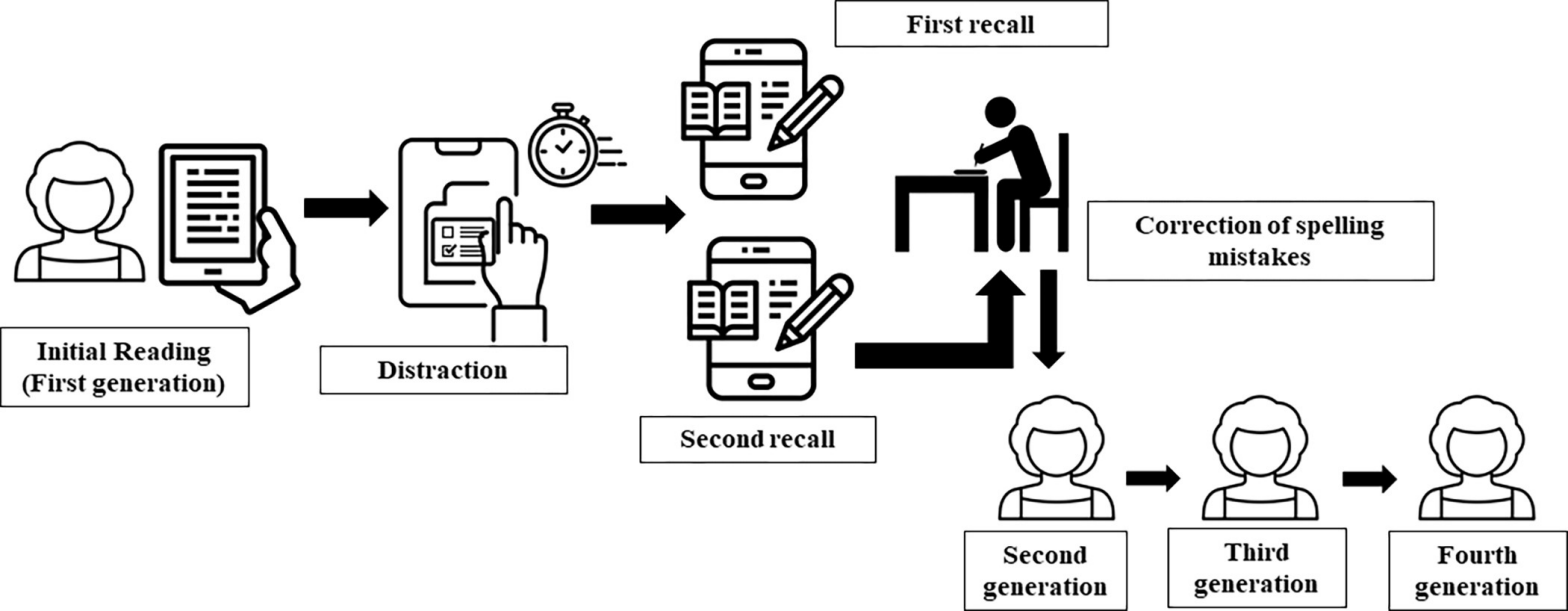
Representation of the information transmission experiment through a linear transmission chain. Figure based on the protocol adapted from Jiménez et al. [31].

Following this, the experiments differ because, after this recall activity, the participant will receive a further instruction: “*Now*, *please transmit the information you obtained from the reading material to someone else by writing it here*. *You have 10 minutes to complete this activity*.” This transmission activity was conceived with the idea of testing whether what the person remembers at first is what they transmit when requested (Fig 4). At the end of this activity, a self-reporting step will take place followed by a message of acknowledgement and appreciation before being removed from the platform. The product of the information recall will undergo a spelling and grammar check, carried out by the researcher the information will then be directed to the next participant in the chain (corresponding to the 2nd generation). This entire process is repeated until the fourth and final participant in the chain is reached.

### Data exclusion criteria

Data referring to the following will be excluded from the survey:

1. Volunteers who participate but are under 18 years of age.
2. Volunteers who abandon the research before the end of the experiment.
3. Volunteers who request the exclusion of their data from the survey.
4. Volunteers who did not complete the attention test/distraction activity.

### Data analysis

To analyze the recall and transmission of information provided by each model in the texts, we consider the central propositions recalled and transmitted by the participants in each of the conditions presented in the texts. Central propositions are the key points of information in a narrative. In our texts, there are seven propositions for each model presented in each of the stories.

Each proposition was identified by its central subject (Table 2), which enabled the identification of the central propositions that were most commonly remembered by the volunteers and the models to which they belonged.

To determine which information is most likely to be remembered and transmitted, we will consider each proposition as a binary result (whether the participant remembered it or not). We will then perform a multilevel logistic regression, considering the participant as a random effect and as a fixed effect: Hypotheses 1 and 2—type of model (digital influencer, physician, politician, and family member). To test the validity of the statistical models, they will be compared with a null model (which will only consider the effect of grouping by participants using the X^2^; test for model adjustments, through the ANOVA function, and the maximum likelihood estimate. The tests will be performed in an R environment [32] using the package lme4 [33].

## Supporting information

S1 File Texts for the experiments. (DOCX)

S2 File Sociodemographic questionnaire used during the distraction stage. (DOCX)

S3 File Distraction activity. (DOCX)

S4 File Self-report questionnaire used at the end of each experiment. (DOCX)

S1 Annex Texts for the experiments. (DOCX)
