# Supplementary material for: Does prestige bias influence the recall and transmission of COVID-19-related information? Protocol registration for an experimental study conducted online
Source: PLoS One. 2023 Feb 23;18(2):e0281991. doi: 10.1371/journal.pone.0281991 (PMC9949656; doi:10.1371/journal.pone.0281991)
Supplement: S2 File — (DOCX) [file pone.0281991.s002.docx]

**Supporting Information 2. Sociodemographic questionnaire used during the distraction stage**

| Date of birth: | | State: | | |
| --- | --- | --- | --- | --- |
| Region of residence:   - Urban - Rural | Gender:   - Female - Male - Non-binary - Other | | | Education:   - Elementary school (incomplete) - Elementary school (complete) - High school (incomplete) - High school (complete) - Graduation (incomplete) - Graduation (complete) - Masters (incomplete) - Masters (complete) - Doctorate (incomplete) - Doctorate (complete) |
| Profession: | | | Income:   - < 1 minimum wage - 1 to 2 minimum wages - 2 to 3 minimum wages - > 3 minimum wages | |
| Religion:   - Catholic - Evangelical - Spiritist - Umbanda - Candomblé - Judaica - Atheist - Other - I don’t have one   If other, please specify____________________________ | | | | |
| Political leaning:   - I consider myself a person of the right wing - I consider myself a person of the left wing - I don’t have a defined political position | | | | |
| Do you want to enter your contact details to participate in the final draw?   - Yes - No | | | | |
| If you answered yes, fill in the contact information you would like to make available: | | | | |
