# Supplementary material for: Does prestige bias influence the recall and transmission of COVID-19-related information? Protocol registration for an experimental study conducted online
Source: PLoS One. 2023 Feb 23;18(2):e0281991. doi: 10.1371/journal.pone.0281991 (PMC9949656; doi:10.1371/journal.pone.0281991)

**Supporting Information 3. Distraction activity**

**Please click on the images that have a tree (you have up to two minutes to complete this task):**


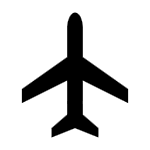

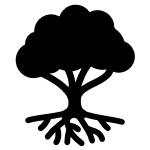

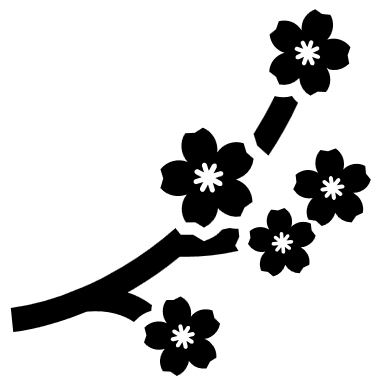

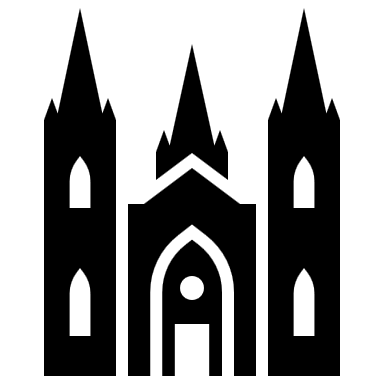

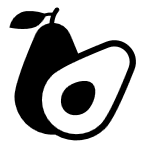

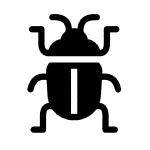

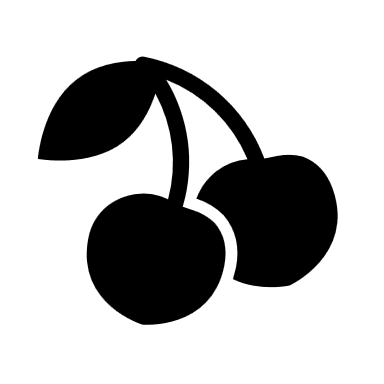

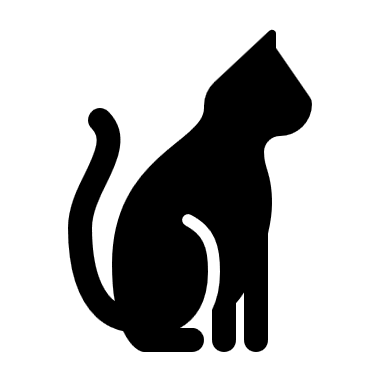

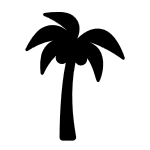

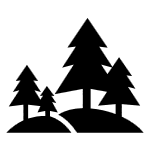

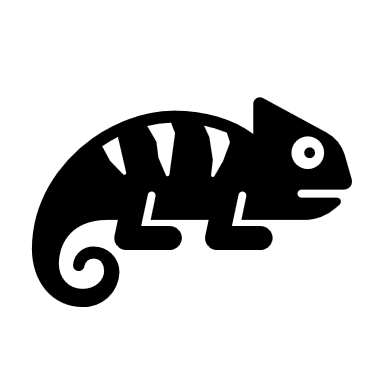

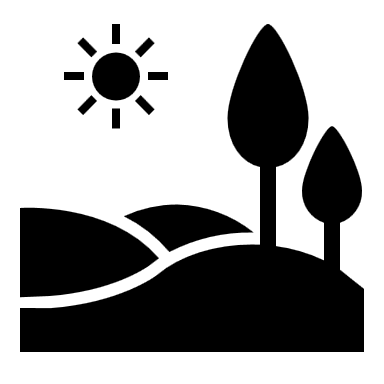

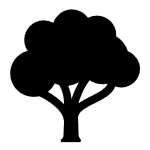

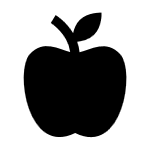

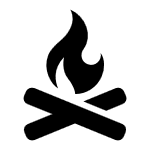

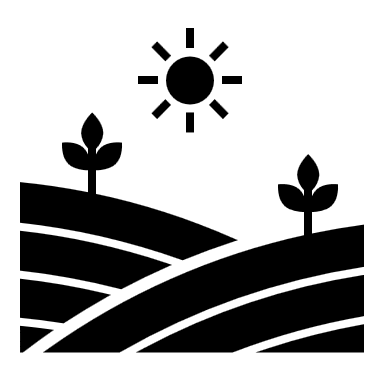

Supplement: S3 File — (DOCX) [file pone.0281991.s003.docx]
