# Supplementary material for: Does prestige bias influence the recall and transmission of COVID-19-related information? Protocol registration for an experimental study conducted online
Source: PLoS One. 2023 Feb 23;18(2):e0281991. doi: 10.1371/journal.pone.0281991 (PMC9949656; doi:10.1371/journal.pone.0281991)
Supplement: S4 File — (DOCX) [file pone.0281991.s004.docx]

**Supporting Information 4. Self-report questionnaire used at the end of each experiment**

| **Self-report questionnaire** | |
| --- | --- |
| Whom do you trust to obtain information from about Covid-19? (you can choose more than one option)   - Health professionals - Politicians I can relate to - Friends - Family - Influencers I follow on the Internet - Religious leaders - Other   Could you give examples of the people you selected? _________________ | What is your primary source of information about Covid-19?   - TV news channels - YouTube videos - Newspapers - Personal pages on Instagram - Personal pages on Facebook - Twitter - WhatsApp - Other   Could you give examples of the vehicles you selected? _________________ |
| Have you been affected by Covid-19?   - Yes - No   If yes, with what intensity?   - Mild symptoms - Moderate symptoms - Severe symptoms | Has any family member been affected by Covid-19?   - Yes - No   If yes, with what intensity?   - Mild symptoms - Moderate symptoms - Severe symptoms - Death |
| Have you been vaccinated?   - Yes - No | |
