# Supplementary material for: Does prestige bias influence the recall and transmission of COVID-19-related information? Protocol registration for an experimental study conducted online
Source: PLoS One. 2023 Feb 23;18(2):e0281991. doi: 10.1371/journal.pone.0281991 (PMC9949656; doi:10.1371/journal.pone.0281991)
Supplement: S1 Annex — (DOCX) [file pone.0281991.s005.docx]

**Texts for the experiments**

1. Prestigious role model

While people were advised to maintain social distance completely during the most critical phase of the COVID-19 pandemic, testing began for a new drug against the novel coronavirus: Postex. As it was being marketed and made available to the population, a digital influencer that you check out every day and admire, with quite a following on social network sites for the content and partnerships they usually endorse, has been quite enthusiastic about the use of this drug. According to him/her, the number of deaths and hospitalizations has dropped considerably since its use has begun, which demonstrates its safe use and that its success rates exceed its failure rates. They have been adamant in advocating for and promoting the use of this drug on their social media.

2. Role model with expertise

While people were advised to maintain social distance completely during the most critical phase of the COVID-19 pandemic, testing began for a new drug against the novel coronavirus: Postex. As it was being marketed and made available to the population, a physician of the local public health authority in their city, who has worked on the front lines in fighting the pandemic, has been quite enthusiastic about the use of this drug. According to him/her, the number of deaths and hospitalizations has dropped considerably since its use has begun, which demonstrates its safe use and that its success rates exceed its failure rates. They have been adamant in advocating for and promoting the use of this drug on their social media.

3. Political role model

While people were advised to maintain social distance completely during the most critical phase of the COVID-19 pandemic, testing began for a new drug against the novel coronavirus: Postex. As it was being marketed and made available to the population, a politician from your area, who is aligned with your beliefs and your usual voting preferences, has been quite enthusiastic about the use of this drug. According to them, the number of deaths and hospitalizations has dropped considerably since its use has begun, which demonstrates its safe use and that its success rates exceed its failure rates. They have been adamant in advocating for and promoting the use of this drug on their social media.

4. Family role model

While people were advised to maintain social distance completely during the most critical phase of the COVID-19 pandemic, testing began for a new drug against the novel coronavirus: Postex. As it was being marketed and made available to the population, a close relative with whom you have a good relationship has been quite enthusiastic about the use of this drug. According to them, the number of deaths and hospitalizations has dropped considerably since its use has begun, which demonstrates its safe use and that its success rates exceed its failure rates. They have been adamant in advocating for and promoting the use of this drug on their social media.
